# A Novel Mass-Producible Capacitive Sensor with Fully Symmetric 3D Structure and Microfluidics for Cells Detection

**DOI:** 10.3390/s19020325

**Published:** 2019-01-15

**Authors:** Zhaorui Zuo, Kun Wang, Libin Gao, Vincent Ho, Hongju Mao, Dahong Qian

**Affiliations:** 1 School of Biomedical Engineering, Shanghai Jiao Tong University, Shanghai 200030, China; zhaorui.zuo@sjtu.edu.cn; 2 State Key Laboratory of Transducer Technology, Shanghai Institute of Microsystem and Information Technology, Chinese Academy of Sciences, Shanghai 200050, China; wangk@mail.sim.ac.cn; 3 Shanghai Institute of Nutrition and Health; Chinese Academy of Science, Shanghai 200031, China; lbgao@sibs.ac.cn; 4 Richtek Technology Corporation, Hsinchu 30288, Taiwan; vincent_ho@richtek.com

**Keywords:** capacitance sensor, biosensor, biochip, affinity sensor, microfluidics, cell detection, multidimensional data

## Abstract

Affinity biosensors of interdigitated electrodes have been widely used in cell detection. This research presents a mass-producible and disposable three-dimensional (3D) structure capacitive sensor based on the integrated circuit package lead frames for cell concentration detection. The fully symmetric 3D interdigital electrode structure makes the sensor more homogeneous and sensitive. (3-Aminopropyl) triethoxysilane (APTES) and glutaraldehyde are immobilized onto gold-plated electrodes. By overlaying the microfluidic channels on top, the volume of the solution is kept constant to obtain repeatable measured capacitance values. Moreover, using the upgraded reading and writing functions and circular measurement of the E4980A Data Transfer Program, an automatic multigroup test system is developed. It is shown that the cell concentration and capacitance are inversely correlated, and the cell concentration range of 10^3^–10^6^ CFU∙mL^−1^ is achieved. In addition, the rate of capacitance change matches that of state-of-the-art biosensors reported. A program is developed to find the optimal voltage and frequency for linear fitting between the capacitance change and cell concentration. Future work will employ machine learning-based data analysis to drug resistance sensitivity test of cell lines and cell survival status.

## 1. Introduction

Affinity biosensors are based on the interaction between immobilized biological components on the transducer surface and target molecules [1]. Biological elements used in affinity biosensors are typically antibodies, DNA, and receptor proteins [1], to name a few. Biological elements have wide applications, such as antibody-based enzyme-linked immunosorbent assay (ELISA) techniques [2], antimicrobial peptide-based sensor for the early bacteria detection [3], and cell-based sensors employed for testing toxicants [4]. 

However, the use of biological sensing elements is limited by low stability and high cost [5]. More recently, some new “artificial antibodies” biological sensors have been proposed, for instance, molecularly imprinted polymers (MIPs) [6] and nanoMIPs [7] have shown good results. These techniques can be promising in various applications where sensitive, selective, and label-free detection is required, however, their manufacturing processes are complicated. Wannapob, R. et al. [1] was the first to use immobilized 3-APBA on a gold electrode for the affinity detection of bacteria with a reproducible sensor, but the sensitivity, which is the capacitance change rate, is small.

An interdigitated electrode array (IDEA) transducer comprises two coplanar comb-like metal electrodes deposited on an insulating substrate. Conventional IDEA sensors are planar devices with a flat sensor surface [3] and are fabricated in thin-film technology (Al and Ni/Au) on silica glass substrates [8]. Three-dimensional (3D) IDEA devices with insulating barriers separating electrode digits, which considerably enhanced the transducer sensitivity, were also reported [9]. Meanwhile, other electronic-based biosensors such as electrochemical biosensors for cancer diagnostics [10], wireless biosensors [11] and impedimetric biosensors [3] for bacteria detection, and disposable screen-printed biosensors for detection of food allergens [12,13] have shown promising results in respective applications.

This research proposed, for the first time, the use of immobilized (3-Aminopropyl) triethoxysilane (APTES) and glutaraldehyde on a gold electrode for the affinity detection of mouse embryonic fibroblast (MEF) cells. Although these sensors are sensitive, the aldehyde group lacks specificity and will bind to the amidogen on the surface of animal cells. Moreover, we designed a completely symmetric 3D structure capacitance sensor based on package lead frames to obtain better sensitivity, uniformity, and stability. In this research, sensor sensitivity has been studied. This sensor can also be potentially used for drug-resistance sensitivity test of cell lines, cell classification and cell survival status evaluation. Furthermore, this sensor is low cost and mass-producible with high repeatability. During the experiments, thousands of sensors were made and used.

## 2. Material and Methods

### 2.1. Preparation of MEF Cells

MEF cells are commonly used to establish the model of senescence, including DNA damage-induced senescence and reactive oxygen species (ROS)-mediated senescence. MEF cells were isolated from pregnant females at embryonic day 13 (E13) and were cultured in Dulbecco’s modified Eagle’s medium supplemented with 10% fetal bovine serum, 1× nonessential amino acids, penicillin, and streptomycin. When cells grow confluently, target cells were digested by 0.25% trypsin, collected by centrifugation at 3000 rpm for 3 min, resuspended in 1× phosphate-buffered saline (PBS), and counted as 10^6^ CFU∙mL^−1^, 10^5^ CFU∙mL^−1^, 10^4^ CFU∙mL^−1^, and 10^3^ CFU∙mL^−1^.

### 2.2. Sensor Design

In this work, we designed a 3D sensor named biocell. Biocell is a biomolecule capacitive sensor based on semiconductor chip package lead frames and can be integrated with a capacitance-to-digital application-specific integrated circuit (ASIC) in the future. Biocell only needs a few discrete components and is easy to use for biomolecule detection applications. It is mass-producible in a WQFN-16L 3 × 3 mm package, which is suitable for embedding in point-of-care testing device.

**Figure 1:**
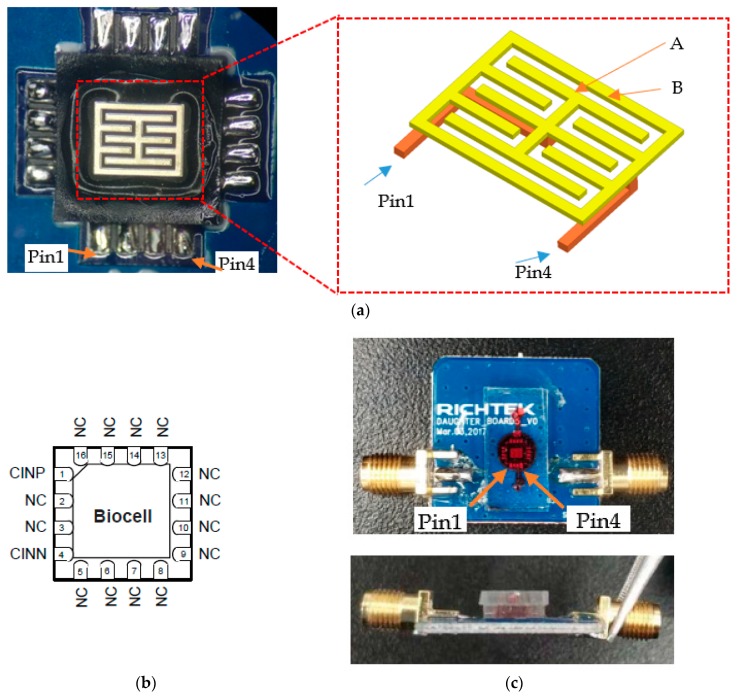
(**a**) Interdigital electrode is fully symmetric to obtain a uniform measured capacitance from cell distribution on electrodes; the biocell metal spacers, A and B, are explained in Section 2.3; (**b**) Pin configurations; (**c**) Red ink fills the microfluidic channel.

The biocell electrodes are basically interdigital electrodes; however, they have outer and inner rings as shown in Figure 1a. The inner ring corresponds to electrode A, while the outer ring corresponds to electrode B. Normally, interdigital electrode sensors have a nonuniform electric field in the outer margin. However, based on fully symmetric 3D structure, electric field near the margin becomes uniform. Inside the sensor, no matter where the cells are attached, the effects on the capacitance are the same. Meanwhile, due to the thickness of the electrodes, the relative area of two electrodes also increases [8], therefore a high change rate of capacitance can be obtained which translates to higher sensitivity.

Biocompatibility is obtained by coating the surface with a layer of gold. Then, biocell is welded onto the printed circuit board (PCB) and connected to the measuring instrument via a sub-miniature A (SMA) interface for high frequency signal input and output. Figure 1b and Table 1 present the pin configurations of biocell, where pin1 and pin4 are multiplexed between the input and output signals. The outline dimension of the sensor is presented in the Supplementary Material (Outline_Dimensions.docx).

### 2.3. Manufacturing Method of Sensor

The standard integrated circuit assembly process [14] is adopted in sensor manufacturing, with an additional layer to isolate the electronic circuit part while keeping the sensing element region open using the open cavity molding method [14]. This open cavity is for putting the target fluid onto the biocell test region. 

**Figure 2:**
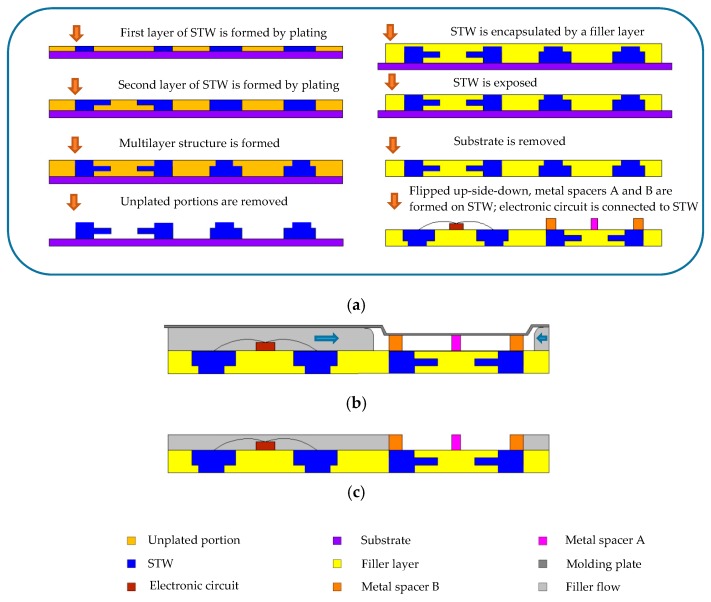
(**a**) Manufacturing flow of biocell; (**b**) Space (i.e., open cavity) is formed between the molding plate and carrier; (**c**) Biocell test region is formed.

In Figure 2a, the metal spacers A and B of the biocell shown in Figure 1a are formed on the signal transmission wiring (STW), and the electronic circuits are mounted on the carrier (surface of the filler layer) to electrically connect the STW. Inside the biocell, the step of mounting the electronic circuits on the carrier is skipped. It is worth mentioning that the electronic circuits can include a controller, an ASIC, or other types of circuits, as required for processing the sensing signals.

Figure 2a shows the manufacturing steps of the carrier wherein the carrier is manufactured by a “molded interconnection system” method [14]. Figure 2b shows that a space (i.e., open cavity) is formed between the molding plate and carrier when the carrier is not yet encapsulated by the package layer. An extrusion of the molding plate is in contact with the outermost portion of metal spacer B (and metal spacer A in this example, but the contact with metal spacer A is not necessary) for defining a limit to block the filler flow such that the filler does not pass beyond metal spacer B to flow into the space between the extrusion and test region. The filler only flows below the molding plate to the space outside the outermost portion of metal spacer B. In Figure 2c, the filler is solidified to form the package layer, and the molding plate is removed. Afterward, the gold layer on the surface of metal spacer A and metal spacer B is formed by electroless plating.

### 2.4. Fabrication of Microfluidic Channels

**Figure 3:**
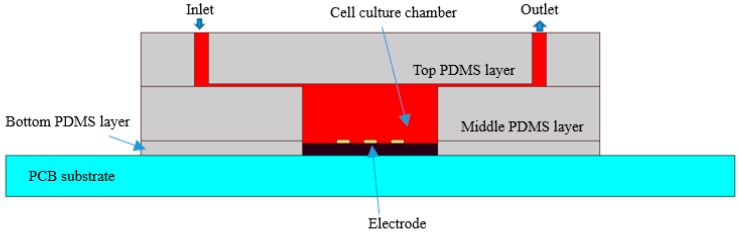
Schematic of microfluidic channels with cross section of closed culturing chamber (drawing not to scale).

The microfluidic channels and chamber are fabricated from polydimethylsiloxane (PDMS) using soft lithography and rapid prototyping techniques. Fabrication of the enclosed PDMS culturing chambers is described thoroughly in Reference [15]. PDMS was chosen owing to its biocompatibility. The enclosed culturing chambers comprised three PDMS layers. As shown in Figure 3, the top layer of 2 mm thickness contains microfluidic channels. The middle PDMS layer of 2 mm thickness contains a cell culture chamber, whose diameter is 6 mm. The bottom PDMS layer of 0.5 mm thickness adheres to the PCB to cover the pins, isolate the solution, and prevent short circuit in the cell solution.

The three PDMS layers were bonded using oxygen plasma treatment, which modifies the exposed surface of PDMS. The bottom layer was immobilized to the PCB substrate by heating it to 95 °C. The actual sensor is shown in Figure 1c. With the microfluidic channels, the volume of cell suspension is maintained while the capacitance of biosensor is uniform and easy to measure. 

### 2.5. Biofunctionalization of Biosensors

To capture cells, the biocell electrode surface must be biofunctionalized. We can bind it to all cells without specific cell selection as long as the surface has an aldehyde group reaction, such as the amidogen group. It contains two layers of chemical molecules of (3-Aminopropyl) triethoxysilane (APTES) and glutaraldehyde.

**Figure 4:**
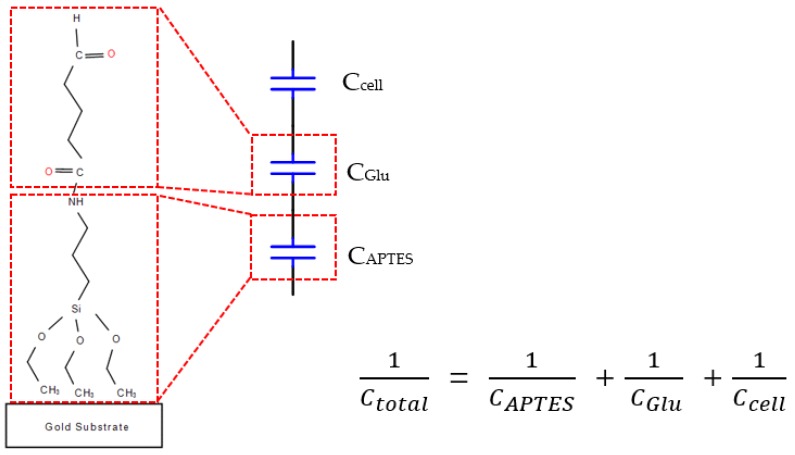
Chemical structure of (3-Aminopropyl) triethoxysilane (APTES) and glutaraldehyde immobilized on the surface. Here, *C*_APTES_ is the capacitance of the APTES layer, *C*_Glu_ is the capacitance of the glutaraldehyde layer, *C*_cell_ is the capacitance of the cell layer, and *C*_total_ is the total capacitance measured at the working electrode solution interface (modified from [17]).

There are three steps of biofunctionalization: (1) Silanization of biosensor surface. First, the gold surfaces of electrodes were cleaned with ethanol, deionized water, acetone, deionized water and piranha solution (3:1, concentrated H_2_SO_4_: 30% H_2_O_2_, *v*/*v*), and deionized water, respectively for 10 min in each step in an ultrasonic cleaner, and then dried using nitrogen gas [16]. Next, the electrodes were cleaned in a plasma cleaner (Model PDC-3XG, Harrick, New York, NY, USA) for 1 min, which is called salinization treatment, and sensors were kept at room temperature in a clean room. (2) Immobilization of APTES. After the plasma process, we immobilized APTES within 5 min with 30 µL 4% APTES concentration of alcoholic solution. After 25 min incubation at room temperature, the electrodes were gently washed with alcohol to remove unbound APTES and then put in a vacuum drying oven. (3) Activating treatment. Thirty µL of 4% glutaraldehyde concentration of deionized water were dropped and after 1h the electrodes were washed with alcohol to remove unbound glutaraldehyde. To this point, the surface of the gold electrode was filled with aldehyde to catch the cells. The assembly sketch map is shown in Figure 4.

### 2.6. Automatic Multigroup Test System

**Figure 5:**
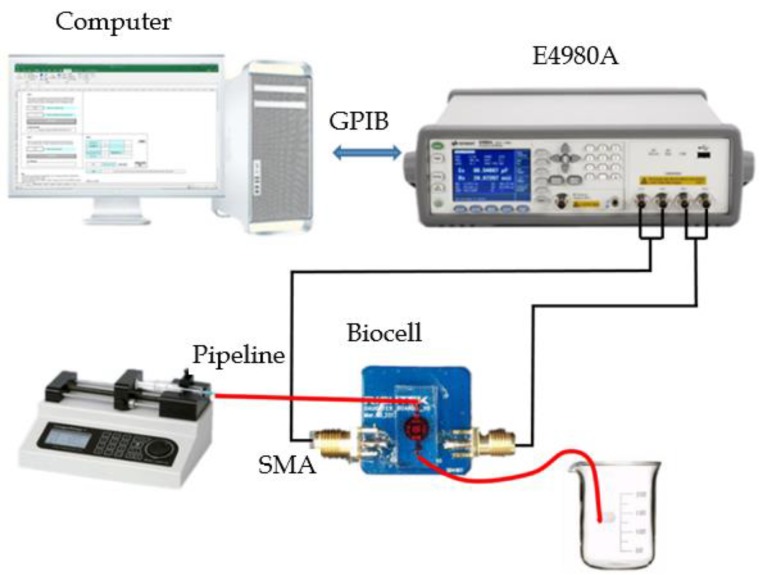
Cell capacitance test system.

An automatic multigroup cell capacitance test system was assembled as shown in Figure 5. The components of the test platform are described below. 

#### 2.6.1. E4980A Data Transfer Program

The Keysight E4980A LCR meter has a seven-digit resolution and its list-scan function allows inputs of up to 201 points of frequencies (20 Hz~2 MHz), test signal levels, or offset levels for automatic measurement. E4980A also supports an automatic testing through a GPIB interface. The official website provides a sample program named E4980A Data Transfer Program (EDTP) in which the PC could read manual measurement data. The software is based on Excel macros, which use Visual Basic for Applications (VBA) programming language, connected to the PC via Virtual Instrument Software Architecture (VISA).

We have upgraded the EDTP for fully automatic multigroup testing by adding some new features: (a) fully remote control and no local operation required (including automatic switching of the display interface); (b) set voltage start value and voltage step to realize voltage scanning when the frequency scan ends; (c) set any measuring frequency point; and d) the delay setting between the measurement groups facilitates a long series of continuous testing at constant voltage and frequency. The upgraded data transfer program (E4980_DataTransfer_64bit_upgraded.xlsm) is given in the Supplementary Material.

#### 2.6.2. Measured Data Format

To stimulate cells with different voltages and frequencies, four sets of data are obtained in each data measurement. The excitation voltages of four data sets are 50 mV, 100 mV, 150 mV, and 200 mV, respectively. In each set of data, there are 20 frequency points at 20, 25, 30, 40, 50, 60, 70, 80, 90, 100, 200, 500, 800, 1000, 2000, 5000, 8000, 10,000, 15,000, and 20,000 Hz.

### 2.7. Capacitance Measurement

The total capacitance is given by the capacitors connected in series as shown in Figure 4 [18], with each capacitor representing the different components on the electrode surface as shown in Figure 4. We found that the change in total capacitance (△*C*) is roughly proportional to the concentration of MEF cells.

The MEF cells were prepared into four concentration groups of 10^6^, 10^5^, 10^4^, and 10^3^ CFU∙mL^−1^. First, a 1× PBS buffer was injected into the cell culture chamber, and the initial capacitance value was recorded when the data became stable after 30 min. Then, a cell suspension was injected and the initial value was recorded. After waiting 1 h for incubation, the data were recorded and the cells with no binding on the surface of the gold electrode were further injected with the 1× PBS buffer and tested again. Finally, a 1× PBS buffer containing 2% 4′,6-Diamidino-2-phenylindole dihydrochloride (DAPI) stain was added and held for 5 min before fluorescence images were collected.

## 3. Results

### 3.1. Capacitance Change of Different Electrode Surfaces

**Figure 6:**
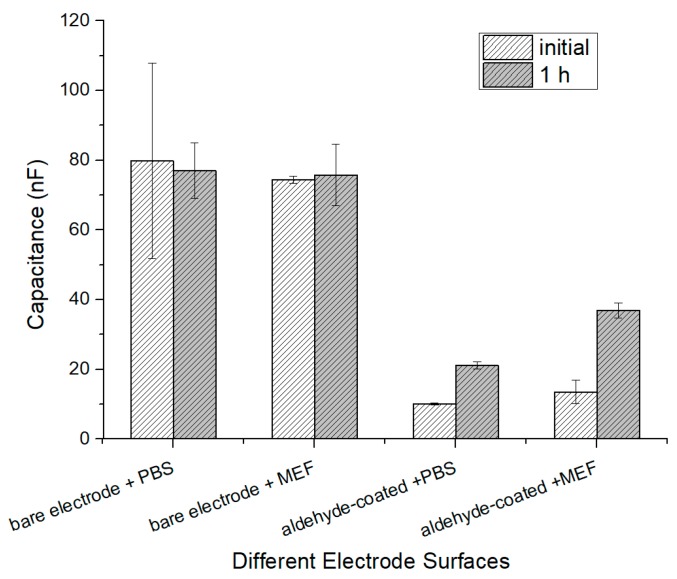
Response from bare electrode and aldehyde-coated electrode in phosphate-buffered saline (PBS) and mouse embryonic fibroblast (MEF) solution.

The capacitance change values of coated electrode and bare electrode in cell solution and PBS solution were compared to verify the effectiveness of the immobilized aldehyde on the electrodes. The different electrodes were measured, as shown in Figure 6, to understand the capacitance change between the coated electrode and the bare electrode in the PBS and MEF cell solution of 10^5^ CFU∙mL^−1^. It is found that the initial capacitance of bare electrode sensors is high and the change rate is low in both PBS solution and MEF solution. Meanwhile, the aldehyde-coated electrode in the PBS has also changed slightly. The last one is the aldehyde-coated electrode in the MEF cell solution of 10^5^ CFU∙mL^−1^, which shows a considerable variation.

### 3.2. Time Response of MEF Cells

**Figure 7:**
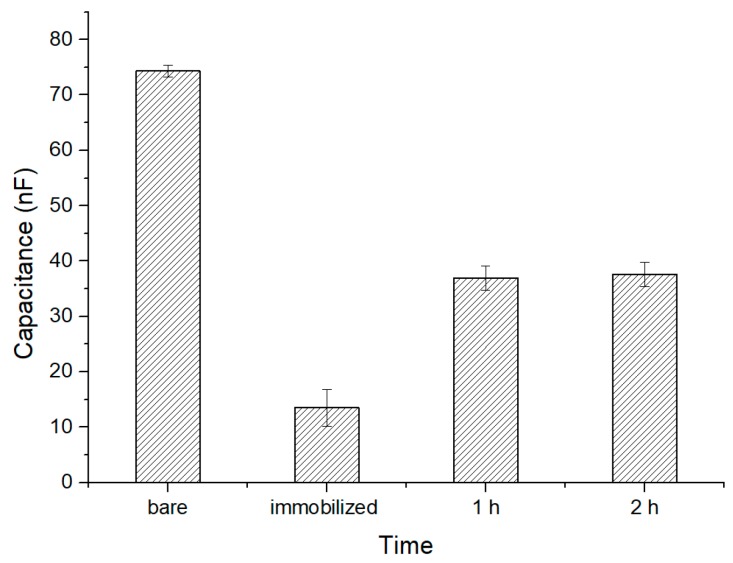
Capacitance values at different periods in a cell solution of 10^5^ CFU∙mL^−1^.

The variation of capacitance with time at 20 Hz frequency and driving voltage of 50 mV was obtained. The capacitance values at different periods in a cell solution of 10^5^ CFU∙mL^−1^ are shown in Figure 7. After (APTES) and glutaraldehyde immobilization, the initial capacitance was small. After 1 h of incubation, the capacitance increased significantly. The capacitance changed only slightly after 2 h, which means that the measurement stabilized after 1 h. It can be observed that the majority of the cells were bonded to the electrode surface after 1 h incubation, and the data after 1 h incubation were recorded in subsequent experiments.

### 3.3. Correlation Between Capacitance, Frequency, and Driving Voltage Range

**Figure 8:**
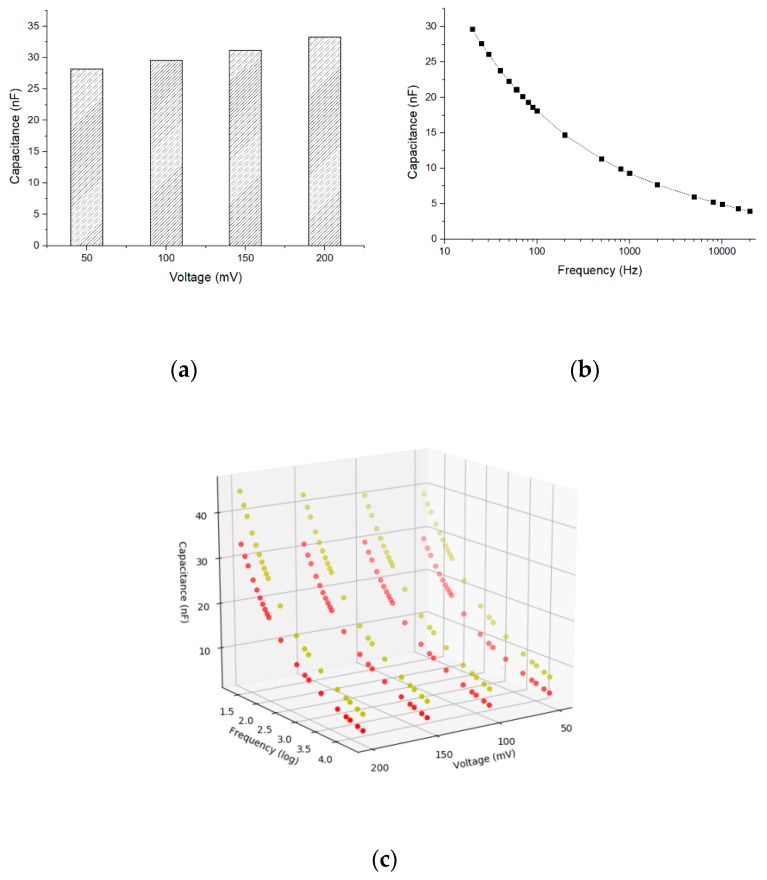
(**a**) Correlation between capacitance and driving voltages at 20 Hz frequency and 10^6^ CFU∙mL^−1^; (**b**) Correlation between capacitance and frequencies at 50 mV driving voltage and 10^6^ CFU∙mL^−1^; (**c**) 3D plot of correlation between capacitance, frequencies, and driving voltages. The red curve is at cell concentration of 10^5^ CFU∙mL^−1^ while the yellow curve is at 10^6^ CFU∙mL^−1^. The frequency axis is in logarithmic scale.

To study the cell response stimulated by different frequencies and driving voltages, we chose the cell concentration of 10^6^ CFU∙mL^−1^ because the larger cell concentration shows a more significant capacitance variation. The measured capacitance is directly related to the driving voltages as shown in Figure 8a, where the frequency is 20 Hz and the capacitance has more variation vs. voltages. At the driving voltage of 50 mV, the correlation between the capacitance and frequency is shown in Figure 8b. The capacitance decreases with increasing frequency. A 3D plot showing the multiple correlations between the capacitance, frequencies, and driving voltages is shown in Figure 8c, where the *x*-axis is the voltage, *y*-axis is the frequency, and *z*-axis is the capacitance. Each voltage and frequency correspond to a capacitance value. In the future, we may be able to classify different cells by machine learning based on multiple input variables of frequencies, driving voltages, and other physical parameters.

### 3.4. Optimal Linear Fitting

**Figure 9:**
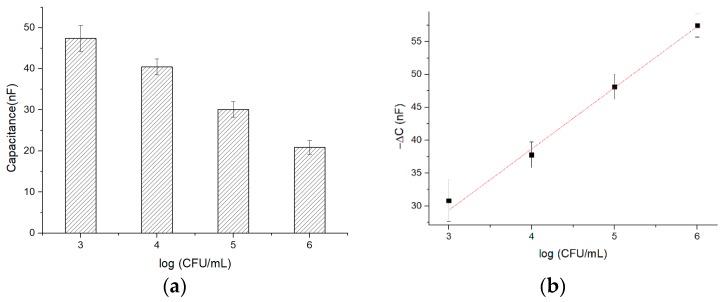
(**a**) Correlation between capacitance and cell concentration at driving voltage of 50 mV and frequency of 20 Hz; (**b**) Optimal linear fitting between −△*C* (*y*-axis) and the logarithmic scale of cell concentration (*x*-axis) is observed in the range of 10^3^–10^6^ CFU∙mL^−1^ at the driving voltage of 50 mV and frequency of 20 Hz.

To evaluate the sensitivity of the 3D sensor in cell detection, a capacitance analysis was performed using MEF cells at different concentrations. The 1 h incubation capacitance (*C*_2_) is shown in Figure 9a; the capacitance has an inverse correlation with the cell concentration ranging from 10^3^ to 10^6^ CFU∙mL^−1^. The lower limit of cell detection of this particular sensor is 10^3^ CFU∙mL^−1^. 

In the cell attachment experiments, the capacitance change (△*C*) was determined by the initial average capacitance of the bare electrodes (*C*_1_) and the 1 h incubation capacitance (*C*_2_). This change is calculated as follows:

(1) $$△C\;=\;C_{2}\;-\;C_{1}.$$

As shown in Figure 8b, we have 80 capacitance points (*C*_2_) under different voltages and frequencies in each cell concentration. To find the optimal voltage and frequency for linear fitting between △*C* and cell concentration, a Python-based program has been developed, and the program (Fit_Linear.py) is given in the Supplementary Material. The program performs linear fitting for all tested voltages and frequencies. Some results of linear fitting are presented in Table 2, and all the 80 fitting results (Linear_Fitting_Result.csv) are given in the Supplementary Material.

In the linear fitting results of △C and the logarithmic scale of cell concentration, *Slope* means the speed of capacitance change (sensitivity of the sensor), and *R^2^* means the degree of linear fitting. We cannot get the maximum *Slope* and *R*^2^ at the same time. Therefore, we define a parameter *Coe* to strike the balance between *Slope* and *R^2^*. The maximum value of *Coe* indicates that both *Slope* and *R^2^* are within the optimal expectation.

The *Coe* is defined as:

(2) $$Coe\;=\;Slope\;+\;10\;\times \;R^{2}$$

It is found that the maximum *Coe* occurs when the voltage is 50 mV and the frequency is 20 Hz. Under these conditions, a linear regression fit (*R*^2^ = 0.9761) of −△*C*, *y*, and cell concentration *x* can be expressed as follows:

(3) y = 9.7627logx − 5.0008

The optimal linear fitting is shown in Figure 9b. 

### 3.5. Biofunctionalization Validation

**Figure 10:**
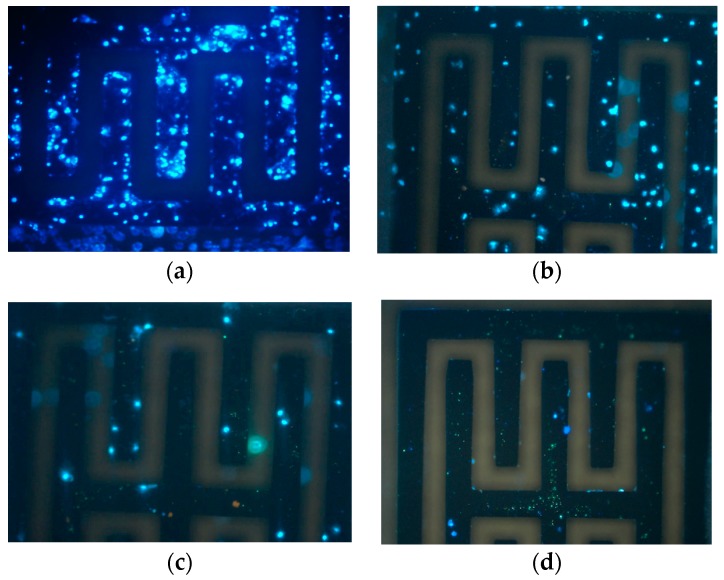
Inverted fluorescence microscope (IFM) images at different concentrations: (**a**) 10^6^ CFU∙mL^−1^; (**b**) 10^5^ CFU∙mL^−1^; (**c**) 10^4^ CFU∙mL^−1^; (**d**) non-biofunctionalized electrodes that were injected with a cell solution of 10^6^ CFU∙mL^−1^.

To further confirm the presence of bacteria owing to biofunctionalization, the electrode surface was visually inspected by an inverted fluorescence microscope (IFM) shown in Figure 10. IFM images are in accordance with the capacitance response measured. No bacterial attachment was observed on non-biofunctionalized electrodes that were injected in a cell solution of 10^6^ CFU∙mL^−1^. In contrast, the presence of cells attached on the surface was clearly observed when the sensors were biofunctionalized with APTES and glutaraldehyde and treated under the same conditions. As expected, cells were not observed in the control sensors as shown in Figure 10d.

## 4. Conclusions

In this study, a novel mass-producible, disposable, and sensitive capacitance biosensor was designed and fabricated. The test region was fabricated using the open cavity molding method [14]. The biosensor’s gold-plated electrodes had a symmetric 3D structure and were coated with APTES and glutaraldehyde to capture cells. We overlaid microfluidics channels to the test region, which contained the gold-plated electrodes to maintain the volume of cell suspension. An automatic data acquisition system was developed to collect multidimensional data and different time series data. First, the capacitance versus voltage was measured at a constant frequency. The capacitance was also measured at a constant voltage and frequency at different time intervals. Our results showed that, the capacitance change (△*C*) after 1 h incubation had a linear correlation with the logarithmic scale of the cell concentration. We then developed a program to select the optimal linear fitting from all the fitting results under different voltages and frequency conditions. The biofunctionalization was verified by fluorescence images of the cells. In the future, this biosensor system can be used for drug-resistance sensitivity test of cell lines, cell classification, and cell survival status evaluation. 

## Figures and Tables

**Table 1:** Pin configurations of biocell.

Pin Number	Pin Name	Pin Function
1	CINP	Sensor input signal
4	CINN	Sensor input signal
2, 3	NC	Not Connected
5–16	NC	Not Connected
Exposed pad	GND	Ground; the exposed pad must be soldered to a large PCB and connected to GND for maximum power dissipation

**Table 2:** Some results of linear fitting.

Voltage (mV)	Frequency (Hz)	Slope	Intercept	*R* ^2^
50	20	9.6727	−5.0008	0.9761
50	25	9.4626	−2.3567	0.9781
50	100	7.7545	14.8953	0.9700
100	40	9.1586	1.4768	0.9758
100	800	4.4053	41.8837	0.7475
150	200	6.9302	21.9895	0.9240
150	5000	2.2606	58.5617	0.5200
200	2000	3.2879	50.6812	0.6155
200	25	10.8184	−14.3831	0.9428

## Supplementary Materials

These are available online at http://www.mdpi.com/1424-8220/19/2/325/s1, CSV-File: Linear_Fitting_Result.csv, VBA-based program: E4980_DataTransfer_64bit_upgraded.xlsm, Python-based program: Fit_Linear.py. Figure S1. The dimension parameters presented in Table S1. Table S1. Outline dimension parameters of biocell.
