# Supplementary material for: A Novel Mass-Producible Capacitive Sensor with Fully Symmetric 3D Structure and Microfluidics for Cells Detection
Source: Sensors (Basel). 2019 Jan 15;19(2):325. doi: 10.3390/s19020325 (PMC6359746; doi:10.3390/s19020325)
Supplement: Supplementary file 1 [file sensors-19-00325-s001.zip › Supplementary File/Outline_Dimensions.docx]

**Outline dimensions of the sensor**


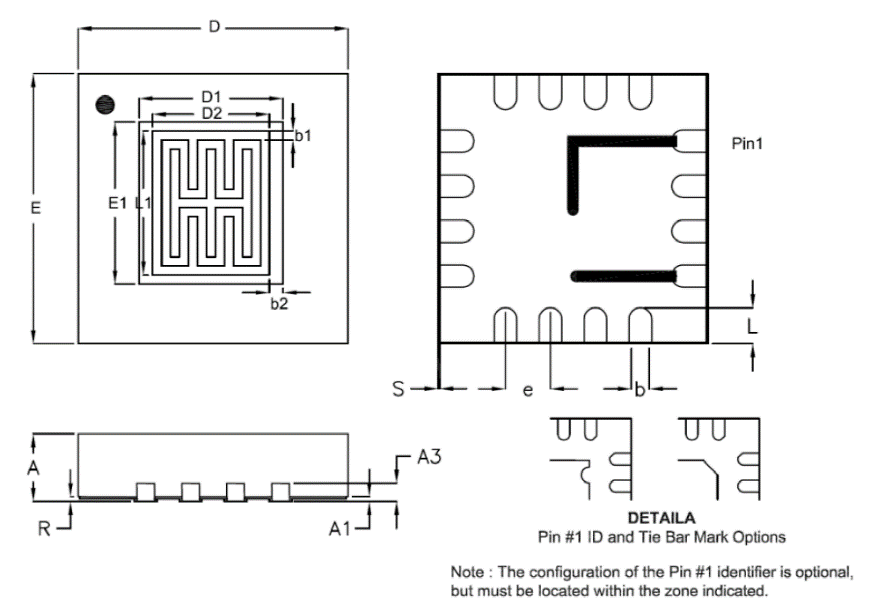


**Figure S1.** The dimension parameters presented in Table S1.

**Table S1.** Outline dimension parameters of biocell

| **Symbol** | **Dimensions in mm** | | **Symbol** | **Dimensions in mm** | |
| --- | --- | --- | --- | --- | --- |
|  | **Min.** | **Max.** |  | **Min.** | **Max.** |
| A | 0.700 | 0.800 | E | 2.950 | 2.950 |
| A1 | 0.000 | 0.050 | E1 | 1.750 | 1.850 |
| A3 | 0.175 | 0.250 | e | 0.500 | 0.500 |
| b | 0.180 | 0.300 | L | 0.350 | 0.450 |
| b1 | 0.050 | 0.150 | L1 | 1.550 | 1.650 |
| b2 | 0.100 | 0.200 | L2 | 1.250 | 1.350 |
| D | 2.950 | 3.050 | R | 0.050 | 0.150 |
| D1 | 1.550 | 1.650 | S | 0.000 | 0.090 |
