# Supplementary material for: A Novel Mass-Producible Capacitive Sensor with Fully Symmetric 3D Structure and Microfluidics for Cells Detection
Source: Sensors (Basel). 2019 Jan 15;19(2):325. doi: 10.3390/s19020325 (PMC6359746; doi:10.3390/s19020325)
Supplement: Supplementary file 1 [file sensors-19-00325-s001.zip › Supplementary File/Software Source Code/E4980_DataTransfer/init/E4980A_DataTransfer_0120.pdf]

Agilent E4980A Precision LCR Meter

E4980A Data Transfer Program
Operation Manual

Rev.01.20

Agilent Technologies

July 2011

Notices

The information contained in this document is subject to change without notice.
This document contains proprietary information that is protected by copyright. All rights are reserved. No part of this document may be photocopied, reproduced, or translated to another language without the prior written consent of Agilent Technologies.
Microsoft®, MS-DOS®, Windows®, Visual C++®, Visual Basic®, VBA® and Excel® are registered trademarks of Microsoft Corporation.
Java® is registered trademark of Sum Microsystems Corporation.

© Copyright 2010 Agilent Technologies

Sample Program

The customer shall have the personal, non-transferable rights to use, copy, or modify SAMPLE PROGRAMS in this manual for the customer’s internal operations. The customer shall use the SAMPLE PROGRAMS solely and exclusively for their own purpose and shall not license, lease, market, or distribute the SAMPLE PROGRAMS or modification of any part thereof.

Agilent Technologies shall not be liable for the quality, performance, or behavior of the SAMPLE PROGRAMS. Agilent Technologies especially disclaims any responsibility for the operation of the SAMPLE PROGRAMS to be uninterrupted or error-free. The SAMPLE PROGRAMS are provided AS IS.

AGILENT TECHNOLOGIES DISCLAIMS ANY IMPLIED WARRANTY OF MERCHANTABILITY AND FITNESS FOR A PARTICULAR PURPOSE.

Agilent Technologies shall not be liable for any infringement of any patent, trademark, copyright, or other proprietary right by the SAMPLE PROGRAMS or their use. Agilent Technologies does not warrant that the SAMPLE PROGRAMS are free from infringements of such rights of third parties. However, Agilent Technologies will not knowingly infringe or deliver software that infringes the patent, trademark, copyright, or other proprietary right of a third party.

1. General information

Overview of the program

This program (a VBA macro for Microsoft Excel®) transfers the list sweep measurement data from the E4980A to a PC.

Supported model and firmware

Model	Firmware
E4980A Precision LCR Meter	Rev. 2.20

Required equipment

- Personal computer
    - * Microsoft Excel® installed
    - * Agilent I/O Libraries Suite 16 or higher installed
- Connection cable (Use either one of the following cables)
    1. USB cable (type mini-B for E4980A side and type A male for PC side, furnished as an E4980A standard accessory)

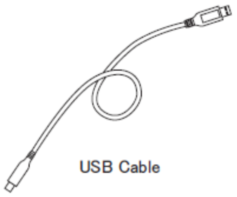

    2. Agilent 82357A or 82357B USB/GPIB Interface

2. Connection configuration through Agilent Connection Expert

1. Run Agilent Connection Expert by double-clicking the icon in the task tray

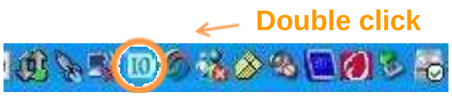

2. Connect the E4980A and the PC using either USB cable or USB/GPIB interface. Then the connection will be automatically configured. After the connection is properly configured, the E4980A appears on “Instrument I/O on this PC” pane of Agilent Connection Expert as shown in the figure below.

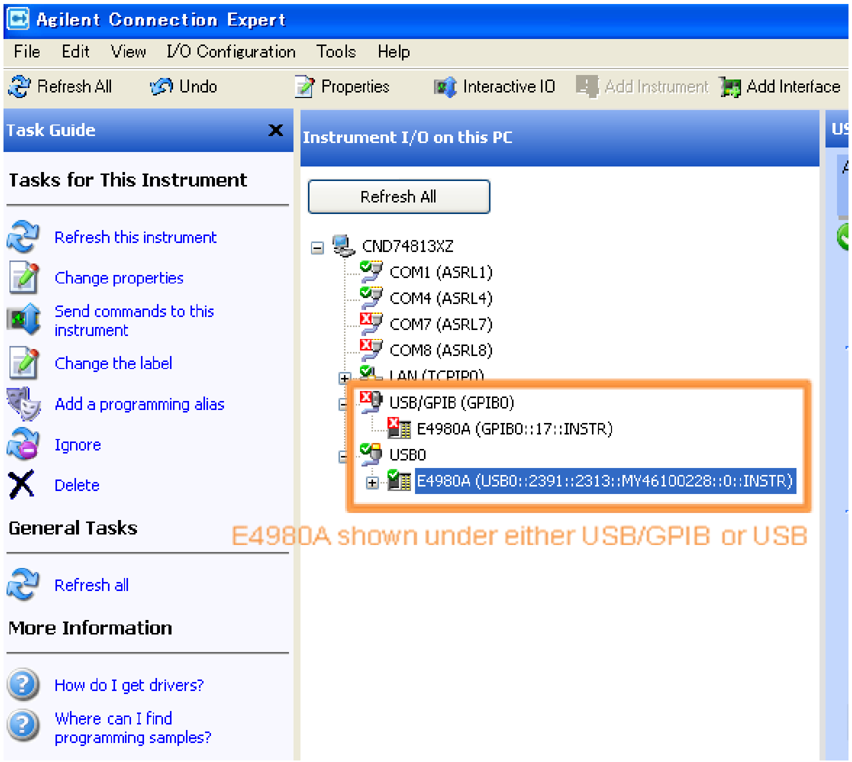

3. How to use the program

1. Set the measurement conditions for the list sweep, then perform the compensation. The E4980A can sweep frequency, test signal level, DC bias signal level, DC source level up to 201 points with the list sweep function by pressing Meas Setup hardkey > LIST SETUP softkey.

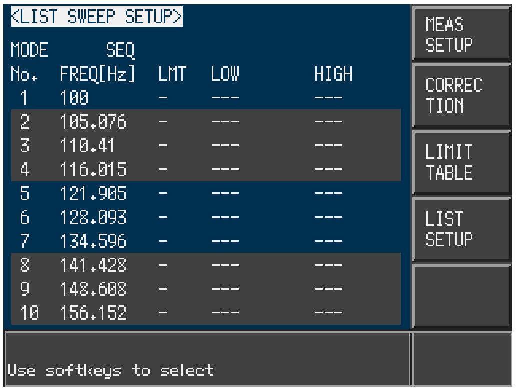

2. Set the trigger to manual mode by pressing Meas Setup hardkey, then cursor down to the TRIG field to select MAN

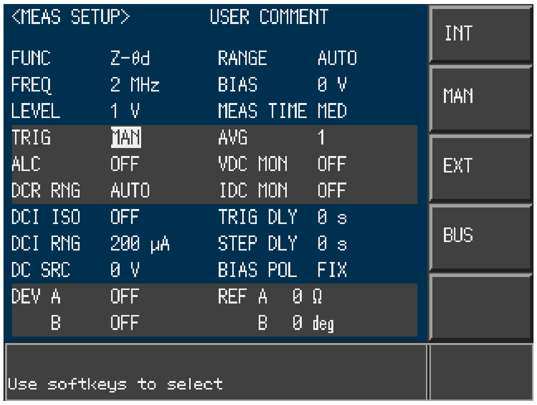

3. Perform the single trigger measurement with the DUT by pressing Display Format hardkey > LIST SWEEP softkey > Trigger hardkey

4. Open “E4980A_DataTransfer_xxxx.xls” on the PC. Since this file contains macros, enable “Macros” to boot the program, then follow the “Step-1” and the “Step-2” in the “Main” sheet.

5. On the newly created sheet, choose the graph parameter to show the graph properly (X/Y-axis are changed to Log format in the example below)

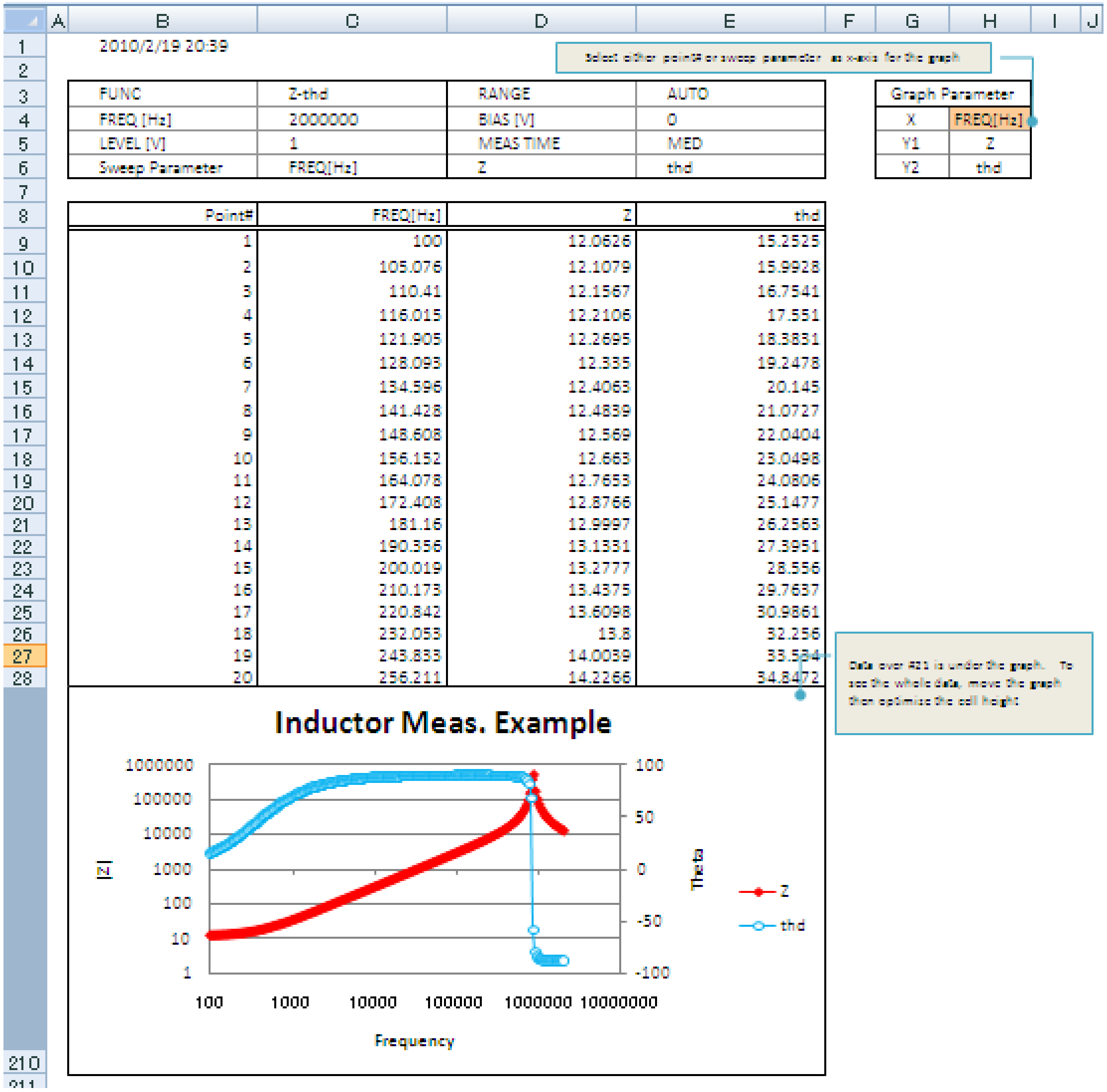

Revision History

Revision	Date	Description
01.20	2011/7/5	- Function added to return to local mode automatically<br>- Function added to show measurement mode as graph title<br>- Evaluated under Windows 7 and Excel 2010 environment<br>- Other minor improvements implemented
01.00	2010/2/22	Initial revision
